# Effects of a novel bacterial phytase expressed in *Aspergillus Oryzae* on digestibility of calcium and phosphorus in diets fed to weanling or growing pigs

**DOI:** 10.1186/2049-1891-4-8

**Published:** 2013-03-05

**Authors:** Ferdinando Nielsen Almeida, Rommel Casilda Sulabo, Hans Henrik Stein

**Affiliations:** 1 Department of Animal Sciences, University of Illinois, Urbana, IL, 61801, USA

**Keywords:** Calcium, Digestibility, Phosphorus, Phytase, Pigs

## Abstract

In 2 experiments, 48 weanling (initial BW: 13.5 ± 2.4 kg, Exp. 1) and 24 growing pigs (initial BW: 36.2 ± 4.0 kg, Exp. 2) were used to determine effects of a novel bacterial 6-phytase expressed in *Aspergillus oryzae* on the apparent total tract digestibility (ATTD) of phosphorus and calcium in corn-soybean meal diets fed to weanling and growing pigs. In Exp. 1 and 2, pigs were randomly allotted to 6 dietary treatments using a randomized complete block design and a balanced 2 period changeover design, respectively. In both experiments, 6 diets were formulated. The positive control diet was a corn-soybean meal diet with added inorganic phosphorus (Exp. 1: 0.42 and 0.86% standardized total tract digestible phosphorus and total calcium, respectively; Exp. 2: 0.32 and 0.79% standardized total tract digestible phosphorus and total calcium, respectively). A negative control diet and 4 diets with the novel phytase (Ronozyme HiPhos, DSM Nutritional Products Inc., Parsippany, NJ) added to the negative control diet at levels of 500, 1,000, 2,000, and 4,000 phytase units (FYT)/kg were also formulated. In Exp. 1, the ATTD of phosphorus was greater (*P* < 0.01) for the positive control diet (60.5%) than for the negative control diet (40.5%), but increased (linear and quadratic, *P* < 0.01) as phytase was added to the negative control diet (40.5% vs. 61.6%, 65.1%, 68.7%, and 68.0%). The breakpoint for the ATTD of phosphorus (68.4%) was reached at a phytase inclusion level of 1,016 FYT/kg. In Exp. 2, the ATTD of phosphorus was greater (*P* < 0.01) for the positive control diet (59.4%) than for the negative control diet (39.8%) and increased (linear and quadratic, *P* < 0.01) as phytase was added to the negative control diet (39.8% vs. 58.1%, 65.4%, 69.1%, and 72.8%). The breakpoint for the ATTD of phosphorus (69.1%) was reached at a phytase inclusion level of 801 FYT/kg. In conclusion, the novel bacterial 6-phytase improved the ATTD of phosphorus and calcium in both weanling and growing pigs. The optimum level of inclusion for this phytase is 800 to 1,000 FYT/kg of complete feed to maximize ATTD of phosphorus and calcium in weanling and growing pigs.

## Background

In feedstuffs of plant origin, phosphorus is present in both organic and inorganic forms. Most of the organic phosphorus in plant ingredients is bound to complex structures called phytate (*myo*-inositol hexakisphosphate), which is the mixed salt of phytate [1]. Phytases hydrolyze phosphomonoester bonds of phytate, which releases bound phosphorus and produces lower forms of *myo*-inositol phosphates [2]. However, digestion of phytate is limited in pigs due to insufficient production of endogenous gastric or intestinal phytases [3,4]. Phytate also has the ability to form calcium-phytate complexes, which renders calcium unavailable for absorption [5,6]. However, adding exogenous phytases to swine and poultry diets improves phosphorus and calcium digestibility and reduces phosphorus excretion [7-9]; and thus, phytase use has become a routine practice. Consequently, exogenous phytases are being developed through genetic engineering based on the gene sequences and protein structures of phytase. The three commonly used phytase feed enzymes are derived from *Aspergillus niger*, which is a 3-phytase and *Peniophora lycii* and *Escherichia coli*, which are 6-phytases [7]. A number of studies compared different sources of exogenous phytase in pigs and observed differences in physico-chemical characteristics [10,11] and efficacy [12,13]. Recently, a novel bacterial 6-phytase (Ronozyme HiPhos, DSM Nutritional Products, Parsippany, NJ) expressed in *Aspergillus oryzae* was developed, but there is no information on the effectiveness of this phytase when fed to pigs. Therefore, 2 experiments were conducted to determine the efficacy of this novel bacterial 6-phytase expressed in *Aspergillus oryzae* on phosphorus and calcium digestibility in corn-soybean meal diets fed to weanling or growing pigs.

## Materials and methods

All experimental protocols used in this study were approved by the University of Illinois Institutional Animal Care and Use Committee. Pigs used in both experiments were the offspring of Landrace boars mated to Large White × Duroc sows (PIC, Hendersonville, TN).

### Animals, diets, and experimental design

For Exp. 1, a total of 48 weanling pigs (initial BW: 13.5 ± 2.45 kg) were blocked by initial BW and randomly allotted to 6 dietary treatments using a randomized complete block design. There were 8 blocks for each collection period. For Exp. 2, 24 growing barrows were used in a 2 period changeover design [14]. In period 1 (initial BW: 36.2 ± 4.0 kg), pigs were blocked by initial BW and randomly allotted to 6 dietary treatments. There were 4 blocks for every collection period. In period 2 (initial BW: 47.3 ± 5.3 kg), the same pigs used in period 1 were allotted in a way that potential residual effects were balanced (i.e., one pig did not receive the same dietary treatment as in period 1, and one dietary treatment did not follow another dietary treatments more than once; [14]). Individual pigs were placed in metabolism cages that allowed for total collection of feces. Each metabolism cage was equipped with a feeder and a nipple drinker.

In each experiment, 6 diets were formulated (Tables 1, 2, 3, and 4). The positive control diet for Exp. 1 and 2 were corn-soybean meal diets formulated to contain calcium and phosphorus levels that meet NRC [15] requirements for weanling (10 to 20 kg) and growing (20 to 50 kg) pigs, respectively. Dicalcium phosphate and limestone were added to the diet to achieve 0.42, and 0.86% standardized total tract digestible phosphorus, and total calcium, respectively, for Exp. 1 and 0.32, and 0.79% standardized total tract digestible phosphorus, and cal-cium, respectively, for Exp. 2. The second diet was the negative control diet formulated to be similar to the positive control diet except that dicalcium phosphate was excluded and replaced with cornstarch. The negative control diet contained 0.16, and 0.48% standardized total tract digestible phosphorus, and total calcium, respect-ively, for Exp. 1 and 0.16, and 0.58% standardized total tract digestible phosphorus, and total calcium, respect-ively, for Exp. 2. In both experiments, 4 additional diets were formulated similar to the negative control diet with the addition of 500, 1,000, 2,000, or 4,000 phytase units (FYT)/kg of the bacterial phytase (Ronozyme HiPhos, DSM Nutritional Products, Parsippany, NJ). One FYT was defined as the amount of enzyme required to release 1 μmol of inorganic phosphorus per minute from sodium phytate at 37°C. Phytase was added to the phytase-supplemented diets as a premix, which was prepared by mixing 3.4% of concentrated phytase (58,700 phytase units/g) with 96.6% cornstarch. All experimental diets were fed in meal form.

**Table 1:** Composition (as-is basis) of experimental diets, Exp. 1

**Ingredient, %**	**Diet**					
	**Positive control**	**Negative control**	**500 phytase**	**1,000 phytase**	**2,000 phytase**	**4,000 phytase**
Ground corn	60.60	60.60	60.60	60.60	60.60	60.60
Soybean meal, (48% CP)	32.00	32.00	32.00	32.00	32.00	32.00
Soybean oil	3.00	3.00	3.00	3.00	3.00	3.00
Limestone	0.90	0.90	0.90	0.90	0.90	0.90
Dicalcium phosphate	1.65	-	-	-	-	-
Cornstarch	-	1.65	1.625	1.60	1.55	1.45
L-lysine•HCL	0.15	0.15	0.15	0.15	0.15	0.15
Salt	0.40	0.40	0.40	0.40	0.40	0.40
Phytase premix ^1^	-	-	0.025	0.05	0.10	0.20
Vitamin and mineral premix ^2^	0.30	0.30	0.30	0.30	0.30	0.30
Mecadox premix ^3^	1.00	1.00	1.00	1.00	1.00	1.00
Total	100.00	100.00	100.00	100.00	100.00	100.00

^1^Ronozyme HiPhos, DSM Nutritional Products, Parsippany, NJ. Produced by mixing 3.4% of concentrated phytase (58,700 units/g) and 96.6% cornstarch.

^2^The vitamin-micromineral premix provided the following quantities of vitamins and micro minerals per kilogram of complete diet: Vitamin A as retinyl acetate, 11,128 IU; vitamin D_3_ as cholecalciferol, 2,204 IU; vitamin E as DL-alphatocopheryl acetate, 66 IU; vitamin K as menadione nicotinamide bisulfate, 1.42 mg; thiamin as thiamine mononitrate, 0.24 mg; riboflavin, 6.58 mg; pyridoxine as pyridoxine hydrochloride, 0.24 mg; vitamin B_12_, 0.03 mg; D-pantothenic acid as D-calcium pantothenate, 23.5 mg; niacin as nicotinamide, 1.0 mg, and nicotinic acid, 43.0 mg; folic acid, 1.58 mg; biotin, 0.44 mg; Cu, 10 mg as copper sulfate; Fe, 125 mg as iron sulfate; I, 1.26 mg as potassium iodate; Mn, 60 mg as manganese sulfate; Se, 0.3 mg as sodium selenite; and Zn, 100 mg as zinc oxide.

^3^The Mecadox premix (Phibro Animal Health, NJ) provided 55 mg per kg of Carbadox to the complete diet.

**Table 2:** Analyzed nutrient composition of diets (as-fed basis), Exp. 1

**Item**	**Diet**					
	**Positive control**	**Negative control**	**500 phytase**	**1,000 phytase**	**2,000 phytase**	**4,000 phytase**
Acid detergent fibre, %	2.70	2.98	2.79	3.04	2.95	2.72
Neutral detergent fibre, %	8.44	9.50	9.84	10.09	8.91	9.55
Phosphorus, %^1^	0.66 (0.42)	0.36 (0.16)	0.36	0.36	0.36	0.35
Calcium, %	0.86	0.48	0.48	0.51	0.56	0.53
Crude protein (CP), %	18.33	17.96	17.24	18.03	19.24	18.27
Dry matter, %	87.42	88.02	87.97	88.08	88.25	88.15
Ash, %	5.74	4.99	4.43	4.27	4.08	4.10
Phytase units	91	80	440	958	1743	3974
Indispensable AA, %						
Arginine	1.26	1.30	1.28	1.19	1.22	1.24
Histidine	0.50	0.53	0.52	0.49	0.50	0.51
Isoleucine	0.80	0.84	0.85	0.81	0.81	0.84
Leucine	1.60	1.66	1.64	1.57	1.57	1.60
Lysine	1.18	1.21	1.20	1.13	1.15	1.20
Methionine	0.29	0.32	0.31	0.29	0.29	0.30
Phenylalanine	0.92	0.95	0.95	0.90	0.90	0.93
Threonine	0.71	0.75	0.71	0.67	0.69	0.69
Tryptophan	0.24	0.24	0.24	0.24	0.23	0.24
Valine	0.92	0.96	0.97	0.93	0.92	0.96
Dispensable AA, %						
Alanine	0.93	0.97	0.94	0.91	0.91	0.93
Aspartic acid	1.92	2.03	1.98	1.87	1.91	1.95
Cysteine	0.31	0.34	0.32	0.29	0.30	0.30
Glutamic acid	3.19	3.32	3.26	3.12	3.14	3.20
Glycine	0.79	0.83	0.81	0.76	0.77	0.79
Proline	0.94	1.10	1.05	1.02	1.04	1.04
Serine	0.83	0.86	0.79	0.76	0.78	0.76
Tyrosine	0.63	0.61	0.61	0.56	0.57	0.57

^1^Values in parenthesis represent the standardized total tract digestible (STTD) phosphorus, which was calculated from the STTD phosphorus values for corn, soybean meal, and dicalcium phosphate presented in Nutrient Requirements of Swine [46].

**Table 3:** Composition (as-is basis) of experimental diets, Exp. 2

**Ingredient, %**	**Diet**					
	**Positive control**	**Negative control**	**500 phytase**	**1,000 phytase**	**2,000 phytase**	**4,000 phytase**
Ground corn	65.80	65.80	65.80	65.80	65.80	65.80
Soybean meal, (48% CP)	29.50	29.50	29.50	29.50	29.50	29.50
Soybean oil	2.00	2.00	2.00	2.00	2.00	2.00
Ground limestone	0.95	0.95	0.95	0.95	0.95	0.95
Dicalcium phosphate	1.05	-	-	-	-	-
Cornstarch	-	1.05	1.025	1.00	0.975	0.95
Salt	0.40	0.40	0.40	0.40	0.40	0.40
Phytase premix^1^	-	-	0.025	0.05	0.075	0.10
Vitamin and mineral premix^2^	0.30	0.30	0.30	0.30	0.30	0.30
Total	100.00	100.00	100.00	100.00	100.00	100.00

^1^Ronozyme HiPhos, DSM Nutritional Products, Parsippany, NJ. Produced by mixing 3.4% of concentrated phytase (58,700 units/g) and 96.6% cornstarch.

^2^The vitamin-micromineral premix provided the following quantities of vitamins and micro minerals per kilogram of complete diet: Vitamin A as retinyl acetate, 11,128 IU; vitamin D_3_ as cholecalciferol, 2,204 IU; vitamin E as DL-alphatocopheryl acetate, 66 IU; vitamin K as menadione nicotinamide bisulfate, 1.42 mg; thiamin as thiamine mononitrate, 0.24 mg; riboflavin, 6.58 mg; pyridoxine as pyridoxine hydrochloride, 0.24 mg; vitamin B_12_, 0.03 mg; D-pantothenic acid as D-calcium pantothenate, 23.5 mg; niacin as nicotinamide, 1.0 mg, and nicotinic acid, 43.0 mg; folic acid, 1.58 mg; biotin, 0.44 mg; Cu, 10 mg as copper sulfate; Fe, 125 mg as iron sulfate; I, 1.26 mg as potassium iodate; Mn, 60 mg as manganese sulfate; Se, 0.3 mg as sodium selenite; and Zn, 100 mg as zinc oxide.

**Table 4:** Analyzed nutrient composition of diets (as-fed basis), Exp. 2

**Item**	**Diet**					
	**Positive control**	**Negative control**	**500 phytase**	**1,000 phytase**	**2,000 phytase**	**4,000 phytase**
Acid detergent fibre, %	2.64	2.63	2.61	2.64	2.67	2.84
Neutral detergent fibre, %	12.11	8.02	8.81	7.71	7.80	8.52
Phosphorus, %^1^	0.56 (0.32)	0.33 (0.16)	0.34	0.34	0.34	0.34
Calcium, %	0.79	0.58	0.59	0.57	0.56	0.54
Crude protein (CP), %	20.45	21.94	21.29	21.00	20.92	20.45
Dry matter, %	88.30	88.36	88.32	88.19	88.21	88.34
Ash, %	4.73	3.98	3.88	4.02	4.02	4.07
Phytase units	39	41	373	984	1773	3681
Indispensable AA, %						
Arginine	1.23	1.22	1.17	1.23	1.23	1.26
Histidine	0.53	0.50	0.50	0.50	0.50	0.51
Isoleucine	0.84	0.81	0.78	0.81	0.82	0.84
Leucine	1.63	1.58	1.54	1.60	1.59	1.62
Lysine	1.08	1.06	1.02	1.07	1.07	1.09
Methionine	0.30	0.29	0.28	0.29	0.29	0.29
Phenylalanine	0.92	0.90	0.86	0.90	0.90	0.92
Threonine	0.70	0.70	0.65	0.70	0.68	0.69
Tryptophan	0.25	0.24	0.25	0.25	0.24	0.25
Valine	0.95	0.91	0.89	0.91	0.94	0.95
Dispensable AA, %						
Alanine	0.94	0.92	0.88	0.92	0.91	0.94
Aspartic acid	1.91	1.87	1.79	1.88	1.88	1.92
Cysteine	0.29	0.30	0.28	0.30	0.30	0.29
Glutamic acid	3.39	3.31	3.18	3.32	3.32	3.37
Glycine	0.79	0.77	0.74	0.77	0.78	0.80
Proline	1.10	1.10	1.01	1.06	1.03	1.07
Serine	0.79	0.80	0.74	0.82	0.76	0.77
Tyrosine	0.57	0.57	0.57	0.59	0.57	0.59

^1^Values in parenthesis represent the standardized total tract digestible (STTD) phosphorus, which was calculated from the STTD phosphorus values for corn, soybean meal, and dicalcium phosphate presented in Nutrient Requirements of Swine [46].

### Feeding and sample collection

All pigs were fed at a level of 3 times their estimated maintenance energy requirement (i.e., 106 kcal ME per kg^0.75^; NRC, [15]) and water was available at all times throughout the experiment. The amount of feed provided daily was divided into 2 equal meals. The initial 5 d were considered an adaptation period to the diet. From d 6 to 11, feces were collected according to the marker to marker approach [16]. Chromic oxide and ferric oxide were used to determine the beginning and the conclusion of collections, respectively. Fecal samples were stored at −20°C immediately after collection.

### Sample analysis and calculations

At the conclusion of each experiment, fecal samples were dried in a forced air oven and ground to pass a 2 mm screen. Fecal samples and diets were analyzed for calcium and phosphorus by inductively coupled plasma (ICP) spectroscopy (method 985.01 [17]) after wet ash sample preparation (method 975.03 [17]). Diets were also analyzed for AA (method 982.30 E (a, b, c) [17]), ADF (method 973.18[17]), NDF [18], DM (method 930.15 [17]), ash (method 942.05 [17]), and CP (method 990.03 [17]). Samples of the diets were sent to DSM Nutritional Products laboratory (Belvidere, NJ) for phytase analysis using the AOAC official method 2000.12 [17].

The apparent total tract digestibility (ATTD) of phosphorus in each diet was calculated according to the following equation:

$$\mathrm{ATTD}\left(\%\right)=\left[\left(\mathrm{Pi-Pf}\right)/\mathrm{Pi}\right]\times 100,$$

where Pi = total phosphorus intake (g) from d 6 to 11 and Pf = total fecal phosphorus output (g) originating from the feed that was provided from d 6 to 11 [19]. The same equation was used to calculate for the ATTD of calcium in each diet.

### Statistical analysis

In Exp. 1 and 2, data were analyzed as a randomized complete block design and as a changeover design [14], respectively, using the MIXED procedure of SAS (SAS Inst. Inc., Cary, NC). In Exp. 1, the model included diet as the fixed effect and block as the random effect. In Exp. 2, the model included diet as the fixed effect and block and period as random effects. Pig was the experimental unit for all analyses. The UNIVARIATE procedure was used to test the normality of the data and to identify outliers. In Exp. 1, there were no outliers. However, 1 outlier was identified in Exp. 2 and was removed from the data set.

For both experiments, contrasts were performed between the positive control and the negative control and the negative control vs. diets with phytase. Orthogonal polynomial contrasts were also conducted to test linear and quadratic responses to the inclusion of increasing levels of phytase to the diets. Appropriate coefficients for unequally spaced concentrations of supplemental phytase were obtained using the interactive matrix language procedure (PROC IML) of SAS. Treatment means were subjected to a least squares broken-line analysis performed using the procedures of Robbins et al. [20] to determine the phytase level needed to maximize ATTD of phosphorus and calcium in weanling and growing pigs. For all statistical tests, an α level of 0.05 was used to assess significance among means.

## Results

### Exp. 1, weanling pigs

**Figure 1:**
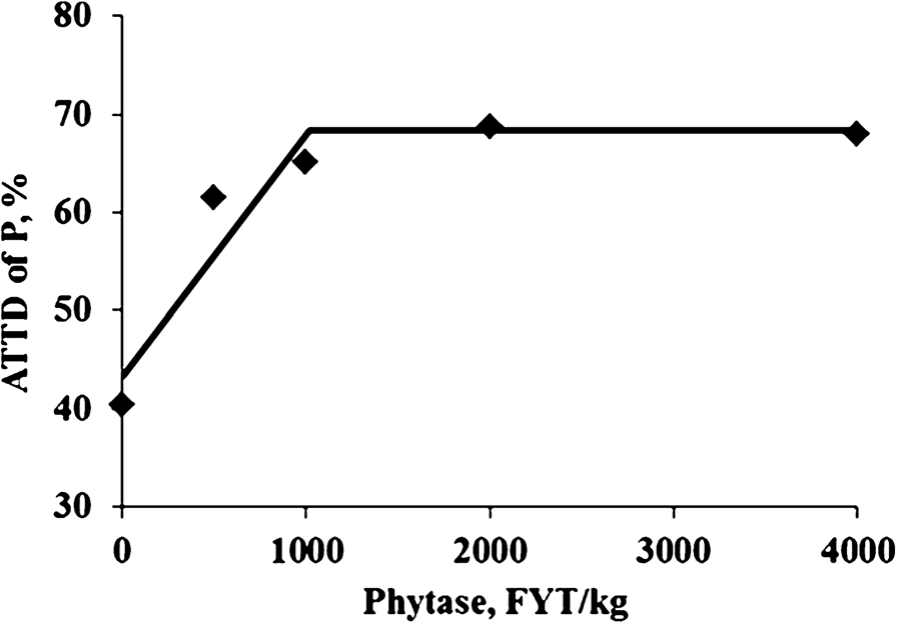
**Fitted broken-line plot of ATTD of phosphorus as a function of dietary phytase level in weanling pigs (Exp. 1) with observed treatment mean values (n = 8 observations per treatment mean).** The minimum dietary phytase level determined by broken-line analysis using least squares methodology was 1,016 FYT/kg (Y plateau = 68.4; slope below breakpoint = −0.025; Adjusted R^2^ = 0.873).

There was no difference in feed intake and fecal output among treatments (Table 5). Phosphorus intake was greater (*P* < 0.01) for pigs fed the positive control diet than for pigs fed the negative control diet, but fecal phosphorus concentration was less (*P* < 0.05) for pigs fed the negative control diet than those fed the positive control diet. Likewise, pigs fed the phytase-containing diets had less (linear and quadratic, *P* < 0.01) fecal phosphorus concentration than pigs fed the negative control diet. The daily phosphorus output was also less (*P* < 0.01) for pigs fed the negative control diet than for pigs fed the positive control diet, and the inclusion of increasing levels of phytase to the negative control diet reduced (linear and quadratic, *P* < 0.01) phosphorus output. The ATTD of phosphorus was greater (*P* < 0.01) for pigs fed the positive control diet than for pigs fed the negative control diet (60.5% vs. 40.5%); however, ATTD of phosphorus increased (linear and quadratic, *P* < 0.01) as phytase was added to the negative control diet (61.6%, 65.1%, 68.7%, and 68.0% for pigs fed diets containing 500, 1,000, 2,000, or 4,000 FYT/kg of phytase, respectively). The amount of phosphorus absorbed was greater (*P* < 0.01) for pigs fed the positive control diet than for pigs fed the negative control diet (2.6 vs. 0.9 g/d). Likewise, the addition of increasing levels of phytase to the negative control diet increased (linear and quadratic, *P* < 0.01) the amount of phosphorus absorbed. The ATTD of phosphorus plateaued at 68.4% which was reached when 1,016 FYT/kg of phytase was added to the diet (Figure 1).

**Table 5:** **Effects of phytase on apparent total tract digestibility (ATTD) of phosphorus and calcium in weanling pigs **^**1**^**, Exp. 1**

**Item**	**Diets**						**SEM**	***P-*value**		***P*-value**^**2**^	
	**Positive control**	**Negative control**	**500 phytase**	**1,000 phytase**	**2,000 phytase**	**4,000 phytase**		**Positive vs. negative**	**Negative vs. phytase**	**L**	**Q**
Feed intake, g/d	645	633	629	646	624	611	18.06	0.65	0.77	0.28	0.69
Phosphorus intake, g/d	4.26	2.28	2.26	2.33	2.25	2.14	0.08	< 0.01	0.68	0.12	0.49
Fecal output, g/d	66.48	58.79	58.06	56.11	57.15	62.61	3.77	0.16	0.94	0.37	0.36
Phosphorus in feces, %	2.53	2.30	1.51	1.46	1.22	1.10	0.07	0.023	< 0.01	< 0.01	< 0.01
Phosphorus output, g/d	1.68	1.35	0.87	0.81	0.71	0.68	0.07	< 0.01	< 0.01	< 0.01	< 0.01
ATTD of phosphorus, %	60.5	40.5	61.6	65.1	68.7	68.0	2.34	< 0.01	< 0.01	< 0.01	< 0.01
Phosphorus absorption, g/d	2.58	0.93	1.39	1.51	1.54	1.46	0.07	< 0.01	< 0.01	< 0.01	< 0.01
Calcium intake, g/d	5.55	3.04	3.02	3.30	3.49	3.23	0.10	< 0.01	0.07	0.07	< 0.01
Calcium in feces, %	2.29	1.86	1.37	1.11	0.94	0.79	0.13	0.019	< 0.01	< 0.01	< 0.01
Calcium output, g/d	1.52	1.09	0.80	0.60	0.52	0.50	0.08	< 0.01	< 0.01	< 0.01	< 0.01
ATTD of calcium, %	72.5	63.9	73.7	81.7	84.8	84.6	2.30	0.012	< 0.01	< 0.01	< 0.01
Calcium absorption, g/d	4.02	1.95	2.22	2.69	2.97	2.74	0.12	< 0.01	< 0.01	< 0.01	< 0.01

^1^Data are means of 8 observations per treatment.

^2^L = linear contrast; Q = quadratic contrast.

**Figure 2:**
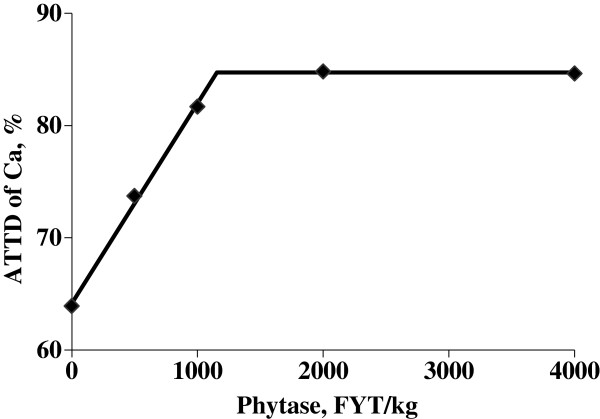
**Fitted broken-line plot of ATTD of calcium as a function of dietary phytase level in weanling pigs (Exp. 1) with observed treatment mean values (n = 8 observations per treatment mean).** The minimal dietary phytase level determined by broken-line analysis using least squares methodology was 1,155 FYT/kg (Y plateau = 84.7; slope below breakpoint = −0.0178; Adjusted R^2^ = 0.997).

Calcium intake was greater (*P* < 0.01) for pigs fed the positive control diet than for pigs fed the negative control diet (5.6 vs. 3.0 g/d). Pigs that were fed phytase containing diets tended (*P* = 0.06) to have a greater calcium intake than pigs fed the negative control diet. Concentration of calcium in feces was greater (*P* < 0.05) for pigs fed the positive control diet compared with pigs fed the negative control diet (2.29% vs. 1.86%); however, pigs fed phytase containing diets had less (linear and quadratic, *P* < 0.01) calcium concentration in feces than pigs fed the negative control diet. The daily calcium output was also greater (*P* < 0.01) for pigs fed the positive control diet than for pigs fed the negative control diet (1.5 vs. 1.1 g/d), but the addition of 500, 1,000, 2,000, or 4,000 FYT/kg of phytase to the negative control diet reduced (quadratic, *P* < 0.01) calcium output to 0.80%, 0.60%, 0.52%, and 0.50%, respectively. The ATTD of calcium was greater (*P* < 0.05) for pigs fed the positive control diet than for pigs fed the negative control diet (72.5% vs. 63.9%), but pigs fed diets containing 500, 1,000, 2,000, or 4,000 FYT/kg of phytase had greater (linear and quadratic, *P* < 0.01) ATTD of calcium than pigs fed the negative control diet (73.7%, 81.7%, 84.8%, and 84.6%). The amount of calcium absorbed was reduced (*P* < 0.01) from 4.0 to 2.0 g/d for pigs fed the negative control diet rather than the positive control diet, but calcium absorption was increased (linear and quadratic, *P* < 0.01) for pigs fed phytase containing diets compared with pigs fed the negative control diet (2.0 vs. 2.2, 2.7, 3.0, and 2.7 g/d). The breakpoint for phytase concentration was reached at 1,155 FYT/kg of phytase, which resulted in an optimal ATTD of calcium of 84.7% (Figure 2).

### Exp. 2, growing pigs

**Figure 3:**
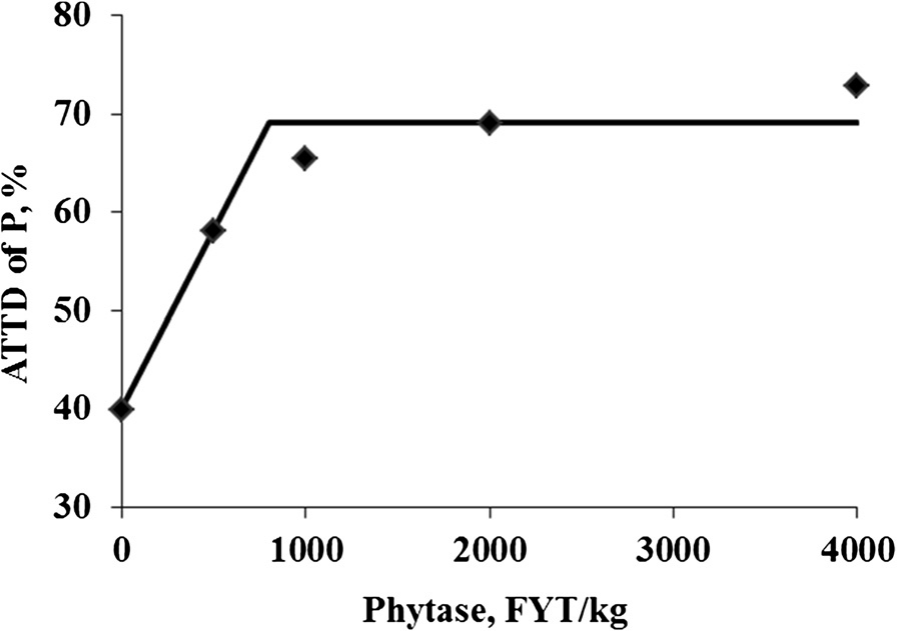
**Fitted broken-line plot of ATTD of phosphorus as a function of dietary phytase level in growing pigs (Exp. 2) with observed treatment mean values (n = 8 observations per treatment mean).** The minimal dietary phytase level determined by broken-line analysis using least squares methodology was 801 FYT/kg (Y plateau = 69.1; slope below breakpoint = −0.036; Adjusted R^2^ = 0.947).

No differences in feed intake were observed among treatments (Table 6). Phosphorus intake was greater (*P* < 0.01) for pigs fed the positive control diet than for pigs fed the negative control diet (8.5 vs. 4.8 g/d) and fecal phosphorus output tended (*P =* 0.08) to be greater for pigs fed the positive control diet than for pigs fed the negative control diet. The phosphorus concentration in feces was less (linear and quadratic, *P* < 0.01) for pigs fed phytase containing diets than for pigs fed the negative control diet. The daily phosphorus output was less (*P* < 0.01) for pigs fed the negative control diet than for pigs fed the positive control diet (2.9 vs. 3.4 g/d). Addition of phytase to the negative control diet reduced (linear and quadratic, *P* < 0.01) daily phosphorus output (2.1, 1.8, 1.5, and 1.4 g/d). The ATTD of phosphorus was greater (*P* < 0.01) for pigs fed the positive control diet than for pigs fed the negative control diet (59.4% vs. 39.8%). Pigs fed phytase containing diets also had greater (linear and quadratic, *P* < 0.01) ATTD of phosphorus than pigs fed the negative control diet (58.1%, 65.4%, 69.1%, and 72.8%). Phosphorus absorption was greater (*P* < 0.01) for pigs fed the positive control diet than for pigs fed the negative control diet (5.1 vs. 1.9 g/d); however, addition of phytase to the negative control diet increased (linear and quadratic, *P* < 0.01) absorption of phosphorus to 3.0, 3.3, 3.5, and 3.7 g/d. The breakpoint for phytase concentration resulted in an ATTD of phosphorus of 69.1%, which was reached when 801 FYT/kg of phytase was added to the diet (Figure 3).

**Table 6:** **Effects of phytase on apparent total tract digestibility (ATTD) of phosphorus and calcium in growing pigs **^**1**^**, Exp. 2**

**Item**	**Diets**						**SEM**	***P-*value**		***P*-value**^**2**^	
	**Positive control**	**Negative control**	**500 phytase**	**1,000 phytase**	**2,000 phytase**	**4,000 phytase**		**Positive vs. negative**	**Negative vs. phytase**	**L**	**Q**
Feed intake, g/d	1521	1460	1506	1497	1476	1476	58.08	0.47	0.67	0.93	0.81
Phosphorus intake, g/d	8.52	4.82	5.12	5.09	5.02	5.02	0.23	< 0.01	0.37	0.83	0.58
Fecal output, g/d	132.86	118.24	116.94	115.95	117.48	123.99	5.48	0.08	0.96	0.36	0.53
Phosphorus in feces, %	2.59	2.44	1.82	1.52	1.31	1.09	0.07	0.17	< 0.01	< 0.01	< 0.01
Phosphorus output, g/d	3.41	2.87	2.12	1.76	1.54	1.36	0.10	< 0.01	< 0.01	< 0.01	< 0.01
ATTD of phosphorus, %	59.4	39.8	58.1	65.4	69.1	72.8	2.25	< 0.01	< 0.01	< 0.01	< 0.01
Phosphorus absorption, g/d	5.10	1.94	3.00	3.33	3.47	3.66	0.24	< 0.01	< 0.01	< 0.01	< 0.01
Calcium intake, g/d	12.02	8.47	8.89	8.53	8.26	7.97	0.36	< 0.01	0.902	0.13	0.84
Calcium in feces, %	2.45	2.33	1.40	1.29	1.22	0.91	0.13	0.54	< 0.01	< 0.01	< 0.01
Calcium output, g/d	3.20	2.74	1.62	1.50	1.46	1.13	0.16	0.07	< 0.01	< 0.01	< 0.01
ATTD of calcium, %	72.9	67.3	81.4	82.6	82.4	85.6	2.05	0.07	< 0.01	< 0.01	< 0.01
Calcium absorption, g/d	8.82	5.72	7.26	7.03	6.80	6.84	0.39	< 0.01	< 0.01	0.38	0.12

^1^Data are means of 8 observations per treatment.

^2^L = linear contrast; Q = quadratic contrast.

**Figure 4:**
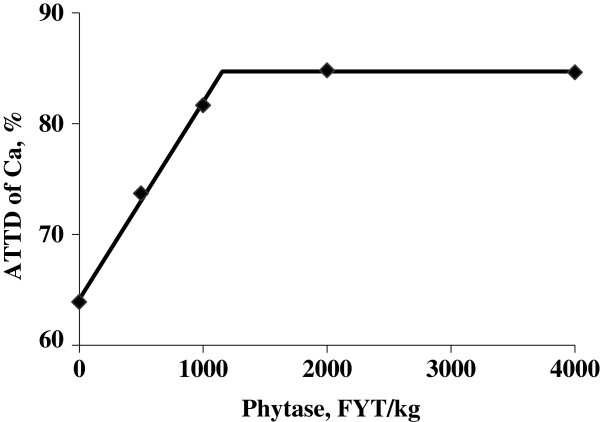
**Fitted broken-line plot of ATTD of calcium as a function of dietary phytase level in growing pigs (Exp. 2) with observed treatment mean values (n = 8 observations per treatment mean).** The minimal dietary phytase level determined by broken-line analysis using least squares methodology was 574 FYT/kg (Y plateau = 83.5; slope below breakpoint = −0.0283; Adjusted R^2^ = 0.958).

Calcium intake was greater (*P* < 0.01) for pigs fed the positive control diet than for pigs fed the negative control diet (12.0 vs. 8.5 g/d). Concentration of calcium in feces was reduced (linear and quadratic, *P* < 0.01) as phytase was added to the negative control diet (2.33% vs. 1.40%, 1.29%, 1.22%, and 0.91%). The daily calcium output tended (*P* = 0.07) to be greater for pigs fed the positive control diet compared with pigs fed the negative control diet (3.2 vs. 2.7 g/d). Addition of phytase to the negative control diet reduced (linear and quadratic, *P* < 0.01) the daily calcium output to 1.6, 1.5, 1.5, and 1.1 g/d. There was also a tendency (*P* = 0.07) for pigs fed the positive control diet to have greater ATTD of calcium than pigs fed the negative control diet (72.9% vs. 67.3%). As phytase was added to the negative control diet, the ATTD of calcium increased (linear and quadratic, *P* < 0.01) to 81.4%, 82.6%, 82.4%, and 85.6%. Calcium absorption was greater (*P* < 0.01) for pigs fed the positive control diet than for pigs fed the negative control diet (8.8 vs. 5.7 g/d). Likewise, pigs fed phytase containing diets had greater (*P* < 0.01) absorption of calcium than pigs fed the negative control diet. For the ATTD of calcium, the breakpoint for phytase concentration was reached when 574 FYT/kg of phytase was added to the diet, which resulted in an ATTD of calcium of 83.5% (Figure 4).

## Discussion

### Effects on phosphorus digestibility

Exogenous phytases are either 3-phytases (EC 3.1.3.8) or 6-phytases (EC 3.1.3.26), which is grouped according to the specific position of the phosphomonoester group on the phytate molecule at which hydrolysis is initiated [21]. Traditionally, phytases of microbial origin are generally considered 3-phytases, whereas phytases from plant origin are 6-phytases [22]; however, 6-phytases from *E. coli, P. lycii,* and the bacterial phytase used in this study are clear exceptions. Thus, previous assumptions regarding the evolutionary distribution of 3- and 6-phytases may be of limited relevance [2]. Exogenous phytases have also been isolated from a variety of sources, expressed in a wide range of hosts, purified, and refolded using various biochemical methods [23]. Depending on the source and expression host, commercially-available phytases have distinct physical and biochemical properties [10,11,24,25] and as a result, they exhibit varying efficacies in pigs and poultry [13,23,26,27]. It is, therefore, important to evaluate the efficacy of new sources of phytase in improving phosphorus utilization for effective use in commercial practice. The phytase used in this study is a 6-phytase from a proprietary strain of bacteria and expressed in a strain of *A. oryzae*. Currently, there are no data on the effects of this novel bacterial 6-phytase on phosphorus utilization by pigs.

In the present study, phosphorus digestibility of the negative control diet was 40.5% and 39.8% for weanling and growing pigs, respectively. These values were within the range determined in previous studies using low-phosphorus, corn-soybean meal-based diets fed to weanling (17.4% to 46.4%; [28-30]) and growing pigs (16.6% to 39.7%; [13,27]). The relatively wide range in phosphorus digestibility of the negative control diets across these studies may be related to the inherent variability of phosphorus digestibility in corn and soybean meal. Previous studies have reported that ATTD of phosphorus in corn ranged from 16.1% [31] to 28.8% [32], whereas in soybean meal, values from 27.6% [33] to 46.5% [34] have been reported. As expected, the phosphorus digestibility values of the negative control diets were less than in the positive control diets. Thus, the amounts of phosphorus absorbed from the negative control diets were reduced compared with the positive control diets, which is mainly an indication of the reduced digestibility of phytate-bound phosphorus in corn and soybean meal compared with inorganic phosphates. Even with the addition of 4,000 FYT to the negative control diet, absorption of phosphorus was not at levels that were similar to the positive control diet. Thus, if one assumes that the positive control diet was at the requirement for phosphorus, this indicates that inorganic phosphorus must be also included in corn-soybean meal diets in combination with phytase.

Values for the ATTD of phosphorus that were observed for weanling pigs fed the diets containing phytase are similar to values reported from previous nursery pig studies in which *A. niger* phytase [28,35] or *E.coli* phytases [9,29,36] were used. Likewise, values for the ATTD of phosphorus obtained in growing pigs fed the phytase containing diets are close to or slightly greater than values reported for pigs fed corn-soybean meal diets containing *E. coli*, *A. niger*, or *P. lycii* phytases [13,37,38]. Thus, the responses observed in this experiment for this phytase, is similar to what has been reported for other commercially-available phytases.

As a result of greater phytate hydrolysis, fecal phosphorus excretion was markedly reduced in weanling and growing pigs fed low-phosphorus diets containing the bacterial 6-phytase compared with pigs fed the positive or the negative control diets. This observation is also in agreement with results of previous experiments [9,28,30,35,38,39]. Thus, the novel 6-phytase used in this experiment is expected to reduce fecal phosphorus excretion to the same degree as other phytases that are currently marketed to the swine industry. Likewise, the increase in the digestibility of phosphorus that was observed by including the novel 6-phytase to the diets is in agreement with results from previous experiments using weanling [9,29,30,36,37] or growing-finishing pigs [13,27].

The use of a broken line model in this experiment may have underestimated the phytase levels that maximises the ATTD of phosphorus and calcium, and a quadratic regression curve could have been a more accurate fit to this data [20]. However, it has been suggested that fitting a quadratic regression curve is preferable when the data consists of at least 4 data points below the breakpoint, which was not the case in this experiment [20]. Results of dose–response experiments using *A. niger* phytase have indicated a curvilinear relationship between phytase level and phosphorus digestibility [40-43], and the maximum response is usually achieved at approximately 1,000 FYT/kg. However, Dungelhoef and Rodehutscord [44] reported that if a fungal phytase is used, improvements in phosphorus digestibility may be minimal if doses greater than 750 FYT/kg of phytase are used. Braña et al. [27] also observed that when using G:F as the response criteria, the maximum response to an *E. coli* phytase was achieved at 738 FYT/kg. Thus, the observation that the response to increasing levels of the bacterial 6-phytase that was used in the present experiments is dose-dependent is in agreement with results obtained with other commercially-available phytases.

### Effects on calcium digestibility

The improvement in calcium digestibility that was observed as phytase was added to the diets is in agreement with previous data [27-29,38] and is likely a result of increased release of calcium during the breakdown of calcium-phytate complexes in the gut. The negative effects of phytate on calcium digestibility may be a result of direct binding of calcium to phytate [8], but phytate may also compromise Na-dependent active transport systems [45]; which may result in reduced calcium digestibility. However, when exogenous phytase is added to the diet and some of the phytates are hydrolized, these negative effects are reduced and calcium absorption is improved.

The linear and quadratic relationship between the level of bacterial 6-phytase in the diet and the improvements in calcium digestibility and fecal calcium output in both weanling and growing pigs is in agreement with data from Jendza et al. [29] and Veum et al. [30]. The current results also indicated that the maximum calcium digestibility was 83.5 to 84.7%, which was obtained with 1,155 and 574 FYT/kg in weanling and growing pigs, respectively.

## Conclusions

Results from the present experiments demonstrate that the novel bacterial 6-phytase expressed in *Aspergillus oryzae* may be used in phosphorus-deficient, corn-soybean meal diets to improve the ATTD of phosphorus and calcium and reduce fecal phosphorus excretion in pigs. Responses of this phytase is similar to or slightly greater than what has been reported for other sources of microbial phytase. The optimum level of inclusion for this phytase is 800 to 1,000 FYT/kg of complete feed to maximize ATTD of phosphorus and calcium in weanling and growing pigs.

## Abbreviations

AA: Amino acids; ADF: Acid detergent fibre; aP: Available phosphorus; ATTD: Apparent total tract digestibility; BW: Body weight; CP: Crude protein; DM: Dry matter; FYT: Phytase units; ICP: Inductively coupled plasma; NDF: Neutral detergent fibre

## Competing interests

Authors have no competing interests.

## Authors’ contributions

All authors equally contributed to this research. All authors read and approved the final manuscript.
